# Coexistence d'un anévrisme sylvien et d'un méningiome intracrânien

**DOI:** 10.11604/pamj.2014.18.112.4081

**Published:** 2014-06-04

**Authors:** Sidi Salem-Memou, Bernard Vallee

**Affiliations:** 1 Service de Neurochirurgie B, de l'Hôpital Neurologique et Neurochirurgical Pierre Wertheimer, Lyon, France

**Keywords:** Anévrisme sylvien, méningiome, angio-Scanner cérébral, MCA aneurysm, meningioma, angio-CT scan

## Image en medicine

La présence concomitante d'un méningiome et d'un anévrisme intracrânien chez un même individu est rare. De nombreuses hypothèses ont été avancées pour soutenir la coexistence des méningiomes et anévrismes. L'augmentation du débit sanguin régional pourrait expliquer une partie des anévrismes comme ceux présents sur les artères nourricières. La coexistence de ces deux lésions présente des implications importantes à la fois sur le plan diagnostic que thérapeutique. Nous rapportons le cas d'une femme âgée de 48 ans, aux antécédents de migraine, qui a été reçu aux urgences pour malaise avec perte de connaissance faisant suite à une céphalée aiguë pariétale droite. L'examen neurologique était normal. Le scanner cérébral révélait une hémorragie sous arachnoïdienne minime avec un anévrysme de la bifurcation sylvienne droite mais également un méningiome de la convexité droite (A, B). L'angio IRM montrait un anévrysme sacciforme au niveau de la bifurcation sylvienne droite présentant un collet d′environ 2,6 mm et un diamètre de 5,5 mm et un processus expansif extra axial à base d′implantation durale visible en regard de la région fronto-temporo-pariétale operculaire droite (D, E, F). Apres une artériographie cérébrale confirmant l'anévrisme sylvien droit au niveau du segment M1 pré bifurcation (C), celui-ci a été embolisé avec succès. La patiente est rentrée à domicile après quelques jours. Elle a bénéficié d'une exérèse de son méningiome un mois plus tard avec des suites simples.

**Figure 1:**
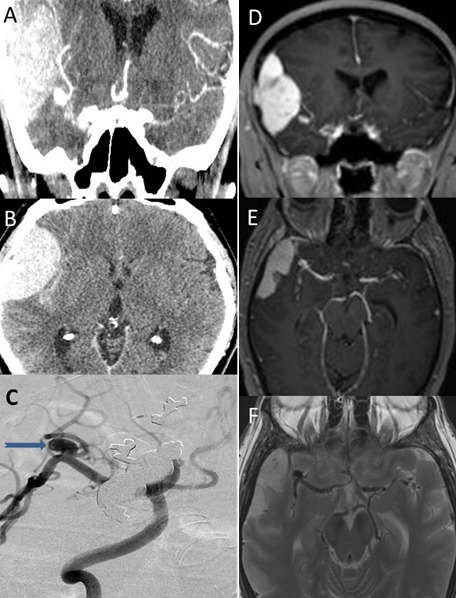
A) Angio-Scanner cérébral en coupe sagittale objectivant un processus expansif droit et un anévrysme de la bifurcation sylvienne droite. B) Scanner cérébral en coupe axiale montrant un processus expansif qui prend le contraste de façon intense et homogène. C) Artériographie cérébrale confirmant l'anévrisme sylvien (flèche) droit au niveau du segment M1 pré bifurcation. D) Angio-IRM en coupe coronale montrant la tumeur et l'anévrisme. E) et F): Angio-IRM en coupe axiale montrant la coexistence de la tumeur et de l'anévrisme

